# Contralateral hyperinflation: Computed tomography demonstration of an unusual complication of unrecognized endobronchial intubation

**DOI:** 10.4103/0972-5229.78229

**Published:** 2011

**Authors:** Jyotindu Debnath, Rajesh Kumar, R. Bala Murali Krishna, Ankit Mathur

**Affiliations:** **From:** Department of Radiodiagnosis, 167 Military Hospital, Pathankot, Punjab, India; 1 Department of Surgery, 167 Military Hospital, Pathankot, Punjab, India; 2 Department of Anesthesiology, 167 Military Hospital, Pathankot, Punjab, India

**Keywords:** Contralateral hyperinflation, endobronchial intubation, computed tomography

## Abstract

Endobronchial intubation (EBI) is an important complication of endotracheal intubation. In a case of unrecognized EBI, usually, the intubated lung gets hyperinflated while the contralateral lung collapses. We report a case of unrecognized right main stem EBI with ipsilateral normal aeration and contralateral hyperinflation detected during computed tomography scan of the chest for trauma work up in a case of severe head injury.

## Introduction

Timely establishment of a patent airway in the form of endotracheal intubation (ETI) is an important component of advanced airway management in critically injured patients. ETI with assisted ventilation can prove life saving during transport of a seriously injured patient to an appropriate trauma center. Overall success rate of pre-hospital ETI carried out by emergency physician/trained paramedics is reported to be more than 90%.[1,2] Amongst the various complications of ETI, unrecognized esophageal and endobronchial intubations are associated with high mortality and morbidity. Undiagnosed main stem endobronchial intubation (EBI) commonly results in hyperinflation of the intubated lung with collapse of the contralateral lung. We report a rare case of right EBI with hyperinflation of the left lung detected during imaging workup computed tomography (CT) scan in a patient of severe head injury.

## Case Report

**Figure 1:**
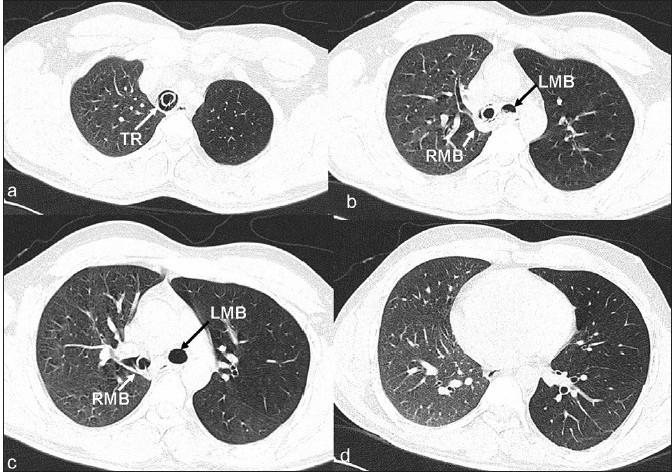
Axial sections at (a) mid thoracic tracheal, (b) carinal, (c) infra-carinal and (d) cardiac levels, respectively. Lung window images depict increased trans-radiance of the lung parenchyma with sparse vascular markings on the left side, suggestive of air trapping. The right lung shows normal CT pattern. Also note mediastinal shift to the right side. Given images also depict presence of ETT in the trachea and right main stem bronchus. TR, trachea; RMB, right main bronchus; LMB, left main bronchus

**Figure 2:**
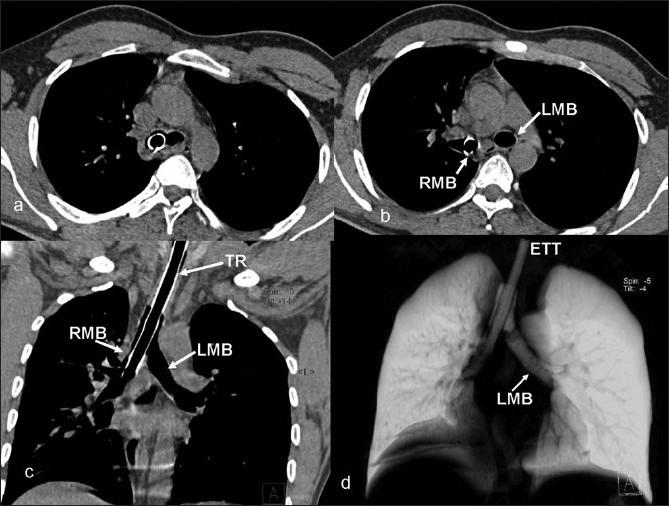
Axial sections (mediastinal window) at (a) carinal and (b) infra-carinal levels confirm the presence of ETT in the right main bronchus. Curved coronal (c) reconstruction (mediastinal window) clearly demonstrates the extent of the ETT in the thoracic trachea and right main stem bronchus. Volume rendered image (d) further confirms findings noted earlier. TR, trachea; RMB, right main bronchus; LMB, left main bronchus; ETT, endotracheal tube

A 26-year-old male was brought to our accident and emergency care center with history of road traffic accident, an hour ago, leading to severe head injury. He was unconscious for almost half an hour immediately after the accident. He was attended by an emergency care physician immediately after the injury and was prophylactically intubated. He was brought to our accident and emergency care center with spontaneous respiration, intermittently manually assisted ventilation with Ambu bag by a paramedic personnel. On arrival at our center, he was found to be comatose with Glasgow coma scale of M_1_ E_1_ V_T_. He had spontaneous breathing with >90% O_2_ saturation at room air and was hemodynamically stable. Besides features of head injury, auscultation of the chest revealed poor air entry on the left side. Though he was hemodynamically stable, he was deeply comatose and hence he was immediately shifted to the CT scan center for an urgent non-contrast CT (NCCT) head to asses the nature and extent of head injury. Since the patient was already in the CT scan center, the surgical specialist requested for an NCCT chest as well to rule out possibility of unrecognized chest injuries, and so, NCCT chest was also done. NCCT scan of the head revealed multiple small (<1 cm) hemorrhagic contusions involving both cerebral hemispheres. CT scan (Sensation 40, Siemens, Erlangen, Germany) of the chest revealed the distal end (approximately 20 mm segment) of the ETT in the right mainstem bronchus. The left lung showed features of hyperinflation with mediastinal shift to the right side. The right lung, however, was normally aerated [Figures 1 and 2]. The ETT, which was lying too low, was pulled out by about 5 cm immediately. Subsequent auscultation demonstrated normal and equal air entry bilaterally.

## Discussion

Although rapid ETI and airway maintenance is considered as life saving in a variety of clinical conditions including critically injured patients, unrecognized misplaced intubations can be more hazardous. Consequences of unrecognized esophageal and endobronchial intubations can be catastrophic. In a prospective study of 149 consecutive out-of-hospital tracheal intubations, Timmermann *et al*, found right main stem endobronchial and esophageal intubations in 16 (10.7%) and 10 (6.7%) patients, respectively.[3] Rolf *et al*, and Ramon *et al*, have reported the incidence of EBI to be 5.1% and 2.4%, respectively.[4,5]

EBI can occur if too long a tube is used and/or inserted beyond the appropriate levels as marked on the ETT. A properly inserted ETT can also get misplaced during head movements and repositioning of the patient as may have happened in our case during transportation to our center. In a case of unrecognized EBI and continued assisted ventilation, contralateral lung collapses while the intubated lung is hyperinflated, predisposing to barotrauma and its sequel.[3] In the case discussed here, the intubated lung was normally aerated as was demonstrated clinically as well as on CT scan, while the contralateral lung was hyperinflated with mediastinal shift to the right side [Figure 1]. The possible explanation for the contralateral hyperinflation rather than collapse as it normally happens could be as follows. Despite right main stem EBI, left lung was partially aerated by collateral flow from the side pores of the distal end of the ETT. Thus, during inspiration, reduced air entry to the left side continued. However, during expiration, there was air trapping in the left lung due to collapse of the right main bronchus over the distal ET tube, blocking the side pores which were the only potential escape route of air from the left lung. Hence, over a period of time, the left lung got hyperinflated. To the best of our knowledge, such a phenomenon of contralateral hyperinflation due to unrecognized EBI with demonstration on CT scan has not been described in the literature.

Timely detection of a misplaced ETT forms an integral part of critical care airway management, may it be in the operation theater or intensive care units or out-of-hospital setting. Although there are a number of means to suspect/detect EBI, a careful clinical examination including auscultation and an appropriate chest radiograph can safely rule out an EBI. CT scan is not routinely required for the diagnosis of the same. Critical care specialists and emergency physicians are generally well versed with clinical and radiological recognition of EBI. Distal end of a correctly placed ETT lies at the level of mid trachea, approximately 3–4 cm proximal to the carina, which would allow flexion and extension of the neck. The minimal safe distance from the carina is considered as 2 cm.[6]

To conclude, our case had an unusual complication of unrecognized right main stem EBI in the form of contralateral hyperinflation as demonstrated on CT scan. Besides individual expertise, following standard precautions and checks during and after ETI can help prevent such complications. Strict adherence to standard trauma radiology protocol including a chest radiograph is essential to detect such complications and take necessary remedial measures in time. Such a protocol helps to obviate the need for sophisticated investigations like CT scan which is of limited availability besides having tremendous radiation burden to the patient
